# Alarming Eosinophilia From Dobutamine Infusion

**DOI:** 10.7759/cureus.12530

**Published:** 2021-01-06

**Authors:** Naji Maaliki, Aleem A Ali, Christopher Izzo, Hamel Patel, Steve Antoine

**Affiliations:** 1 Internal Medicine, University of Florida College of Medicine, Jacksonville, USA; 2 Cardiology, University of Florida College of Medicine, Jacksonville, USA

**Keywords:** eosinophila, dobutamine, cardiogenic shock, heart failure, hyper-eosinophilia syndrome, critical care cardiology, drug-induced eosinophilia

## Abstract

A 49-year-old male with a history of nonischemic heart failure with reduced ejection fraction, hypertension, diabetes was admitted for cardiogenic shock. Treatment started with a high dose of dobutamine infusion. While the patient’s volume status improved, his clinical status declined as he became febrile and hypotensive. He was found to have severe dobutamine-induced eosinophilia, corrected only upon dobutamine cessation and steroid administration. A comprehensive investigation ruled out other potential etiologies. Peripheral eosinophilia is a rare adverse effect associated with dobutamine, leading to a significant deterioration in already decompensated patients.

## Introduction

Peripheral eosinophilia is a rare disease process with multiple etiologies. Among the culprits is dobutamine, which can discretely but surely elevate the eosinophil count to massive levels. Dobutamine-induced eosinophilia can be detrimental, leading to significant hemodynamic compromise. We present a 49-year-old patient admitted for cardiogenic shock who was started on dobutamine, later to develop severe eosinophilia requiring treatment escalation. This case underlines the importance of recognizing the potential side-effects of dobutamine, which can lead to deterioration in unsuspecting individuals.

## Case presentation

**Figure 1:**
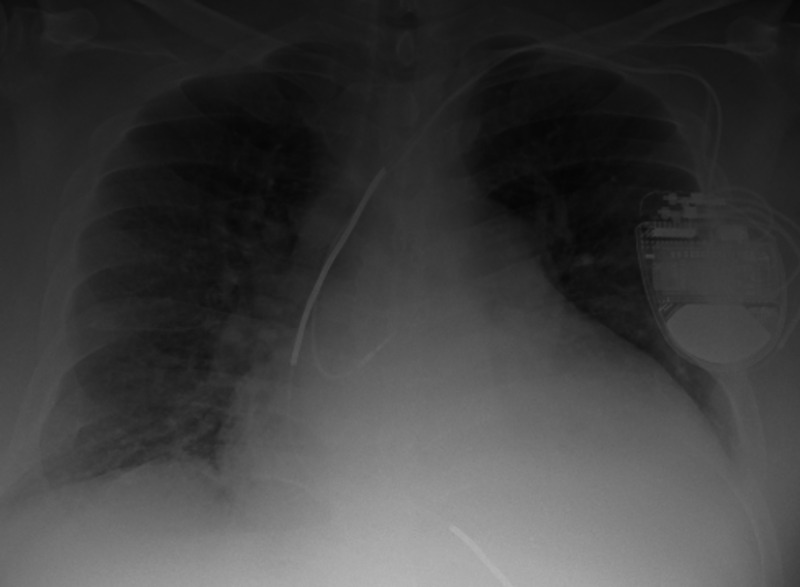
Chest X-ray demonstrating bilateral pulmonary edema and cardiomegaly supportive of heart failure.

**Figure 2:**
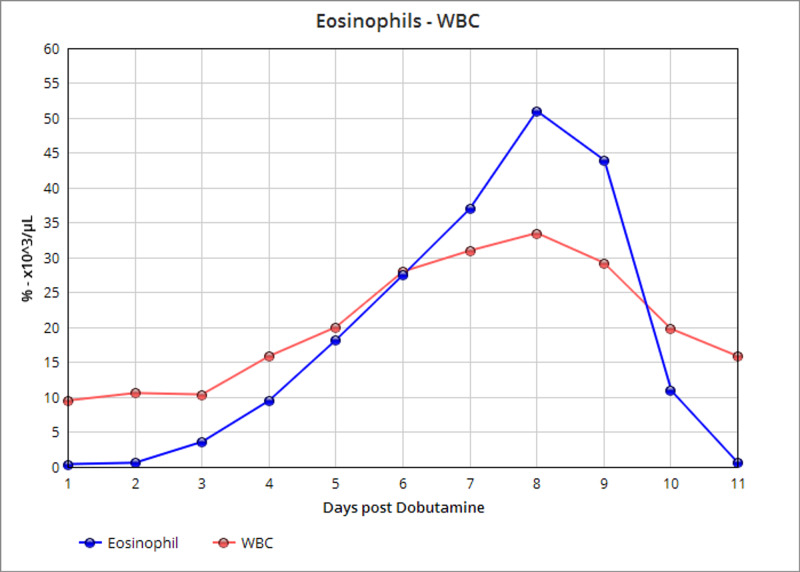
Eosinophil percentage and WBC increase during dobutamine infusion (stopped after day 8). WBC: white blood cell count.

A 49-year-old male with a past medical history of nonischemic heart failure with reduced ejection fraction, hypertension, diabetes presented to our hospital for shortness of breath. He was tachycardic, hypotensive, and afebrile. Physical exam revealed elevated jugular venous pressure, bilateral rales, abdominal distention, and lower limb edema with cold extremities. A chest X-ray confirmed cardiomegaly with bilateral lower lobe infiltrates, and NT-Pro BNP was 8774 pg/ml (Figure 1). White blood cell (WBC) count was nonsignificant at 7.53 x10^3^/μL with 0.7% eosinophils. He was placed on a continuous dobutamine infusion at 10 mcg/kg/min along with a furosemide infusion. While the patient's volume status began to improve, the WBC count increased after a few days. He became febrile, hypotensive, with increased malaise and confusion. Physical exam was unaltered, and pan-cultures were positive for urinary Citrobacter Freundii treated with Nitrofurantoin based on sensitivities, but the WBC continued to elevate. Broad-spectrum antibiotics were initiated with meropenem and vancomycin, which did not affect the WBC count, now at 28.24 x10^3^/μL. Surprisingly, the eosinophil percentage count was remarkably high, progressively increasing from 7.3% to 33% (Figure 2). The other cell lines on complete blood count were normal. The exam did not reveal any skin rashes, new cough, wheezing, diarrhea, vomiting, or muscle pain. The patient had not traveled outside of the city in years, did not indulge in outdoor activities, and claimed to eat out of home rarely. Infectious workup, including stool ova and parasites, Strongyloides, and fungal serologies, was negative. Further investigations with an autoimmune workup, serum protein electrophoresis, troponins, and creatinine kinase levels were negative as well. Immunoglobulin E was elevated, which raised concern for a hypersensitivity reaction. Due to progressive hemodynamic instability, hydrocortisone and norepinephrine were initiated, along with a switch to Aztreonam monotherapy. The eosinophil count continued to elevate, now at the peak of 51% of 33.54 x10^3^/μL. Eventually, dobutamine was switched to milrinone, and the eosinophil count began to decrease the day after. Over the ensuing days, the patient's clinical status also improved, as he was weaned off hemodynamic support along with eosinophil count normalization.

## Discussion

Peripheral eosinophilia is defined as an absolute eosinophil count of more than 500/µL, with severe eosinophilia characterized by levels above 5,000 µL [1]. Clinical manifestations include asymptomatic cases, hypersensitivity reactions such as airway compromise and skin rashes, and sepsis-like presentations with end-organ damage such as myocarditis. The etiology can range from allergic, infectious, or idiopathic processes [2]. In our case, eosinophilia occurred during the hospital stay, which makes a parasitic infection less likely. Nevertheless, he had not traveled years before admission and lived in the city with no outdoor activities. The negative stool ova and parasite test, Strongyloides, fungal serologies, and conventional infectious workup supported this. The elevated IgE levels alluded to a hypersensitivity reaction. A review of medications revealed minimal possible causes (Table 1). Nitrofurantoin rarely causes eosinophilia and follows an insidious pattern with pulmonary involvement when it does [3]. The sudden onset and lack of respiratory disease make this unlikely. While beta-lactams were used, the onset of eosinophilia had occurred well beforehand. Furosemide is rarely associated with Drug Reaction with Eosinophilia and Systemic Symptoms (DRESS) syndrome, but the eosinophilia was resolved while the patient was still receiving the medication. Additionally, DRESS was unlikely due to the eosinophilia's rapid onset and resolution, the lack of rash, lymphadenopathy, and transaminitis. The self-limiting nature of the process also ruled out malignancy, and the negative workup lowered the likelihood of an autoimmune process. The hematology team had recommended a bone marrow biopsy that was later canceled due to the normalization of levels.

**Table 1:** Medications commonly associated with eosinophilia [9].

Medication class	Medications
Antibiotics	Penicillin derivatives, Cephalosporins, TMP-SMX, Nitrofurantoin, Linezolid, Dapsone, Isoniazid
Antiepileptics	Phenytoin, Carbamazepine, Lamotrigine, Phenobarbital
Chemotherapeutics	Methotrexate, Bleomycin
Miscellaneous	Amiodarone, Celecoxib, Fluoxetine, Allopurinol, Captopril

Eosinophilia has been associated with dobutamine, albeit with different manifestations. The mechanism of dobutamine-induced eosinophilia is still not fully understood but could be related to an allergic hypersensitivity reaction. Dobutamine infusions commonly contain sodium bisulfite, which is a known allergen [4]. The eosinophilia usually correlates with infusion duration, with short-term treatment associated with local and immediate hypersensitivity reactions, and long-term treatment causing severe peripheral eosinophilia [5]. Additionally, dobutamine contains many other compounds that may lead to hypersensitivity reactions [6]. As mentioned earlier, symptoms vary, ranging from subclinical and local skin irritations to "full-blown" systemic inflammation [7]. Eosinophilic myocarditis is a possible manifestation, presumably due to the heart's abundance of beta-1 receptors, leading to significant hemodynamic distress [7]. The diagnosis is made by linking the onset of eosinophilia with the timing of dobutamine infusion and ruling out other causes, confirmed by an endomyocardial biopsy when myocarditis is suspected [8]. Treatment is through dobutamine cessation, followed by steroid therapy in refractory cases [5].

## Conclusions

In this case, dobutamine was deemed to be the potential cause of eosinophilia. While an endomyocardial biopsy for confirmation was not done, the clinical correlation makes this association possible. Dobutamine-induced eosinophilia is a rare cause of clinical compromise in unsuspecting patients. There could be many reasons for the deterioration in cardiogenic shock cases, but attention should be given to dobutamine's uncommon hematologic side-effects.
